# Grand Challenges in Immunological Memory

**DOI:** 10.3389/fimmu.2017.00385

**Published:** 2017-04-05

**Authors:** Scott N. Mueller

**Affiliations:** ^1^Department of Microbiology and Immunology, The University of Melbourne, at the Peter Doherty Institute for Infection and Immunity, Melbourne, Australia

**Keywords:** immunological memory, lymphocytes, MHC tetramers, epigenetics, transcription factors, infection, tissue-resident memory T cells, human immunology

Immunological memory is a central feature of the immune system that can provide long-lived, specific protection against previously encountered diseases. Despite our increasingly complex understanding of the cellular players, we still do not fully understand the mechanisms underlying immunological memory, and therefore, how best to induce effective memory responses to many infectious diseases.

In the past two decades, significant progress has been made revealing the complexity of lymphocyte populations induced by infections. The use of T-cell receptor transgenic mice in adoptive transfer experiments and major histocompatibility complex tetramer reagents has enabled immunologists to track T cell responses in mice and humans from the naïve lineage through to memory and during recall of resting memory populations (1, 2). This has provided a wealth of information about T cell specificity, homeostasis, trafficking, and facilitated the definition of multiple subsets of memory T cells. At least three main subsets of memory T cells have been defined, including central memory (T_CM_), effector memory (T_EM_), and tissue-resident memory (T_RM_) T cells (3). Antigen inexperienced CD8 T cells with a memory phenotype have also been described and may contribute to immunity. As knowledge of T cell responses has advanced, our understanding of memory B cell and long-lived plasma cell formation within tissues has come a long way. With the application of B cell receptor transgenic mice and increasingly sophisticated tools to analyze responses in humans, new research is adding to a wealth of information on humoral immunity and memory to pathogens and vaccines (4).

A challenge for the field moving forward will be to better define memory lymphocyte subsets on the basis of function as well as phenotype, the relationships between subsets, and importantly how each correlate with protective immunity. This will be particularly crucial in humans, where T cell responses are commonly only examined in peripheral blood, whereas memory T cells, especially T_RM_ cells, are highly compartmentalized within non-lymphoid tissues (5). Defining immunological memory in human tissues and determining how to induce circulating and tissue-resident lymphocytes through vaccination is an outstanding challenge for the field.

With technological advancements in intravital imaging and cell tracking, the cellular interactions involved in the activation of T and B cells to vaccination and infection are becoming clearer (6). It will be important to better define the cellular interactions in lymphoid and non-lymphoid tissues that underlie the development and homeostasis of memory lymphocyte subsets. Both T_RM_ cells and memory lymphocytes that transit from blood to tissues and back again are influenced by tissue microenvironments (7). As such, an important goal should be to define which signals imprint lymphocytes with the ability to develop into different memory subsets and that direct cells to reside in specific tissues and organs. This will be a vital step to combat many diseases, where mucosal, cutaneous, or nervous tissues serve as primary pathogen entry points. In addition, understanding the influence of the microbiome on the formation and maintenance of immunological memory is lacking but could hold substantial promise (8). Recent work highlighting differences between the memory compartments in experimental SPF mice with humans or outbred mice housed under non-SPF conditions (pet store mice) also implicate a need to include models of immunological memory that better reflect that in humans (9).

Another area where significant progress continues to be made is in dissection of the molecular pathways required for memory lymphocyte development and function (10). Identification of key transcription factors and transcriptional networks will help in defining how we can better guide memory responses *via* vaccines and adjuvants. Likewise, how epigenetic modifications are induced and maintained within memory lymphocytes remains to be fully elucidated (11), but advances in the field are making such experiments with small populations of memory cells considerably easier. It will then be critical to determine whether pharmacological interventions to manipulate epigenetic pathways, including DNA methylation and histone acetylation, could provide therapeutic avenues to complement vaccination. In addition to this, we must consider the long-term changes to the metabolism of memory cells that can promote their functions and possibly also persistence (12). Are metabolic changes attuned to tissue microenvironments and can we manipulate this for therapeutic gain?

How memory cells perform immunosurveillance functions and then rapidly respond upon antigen encounter within tissues remains poorly understood and requires further study. Memory T and B cell subsets each contribute to protection from reinfection, yet the relative roles of distinct subsets in the context of different infections and tissues are not clear. The ability to analyze single cells at the genomic and proteomic level may prove useful in this regard, as advanced tools to analyze “omics” data and detect differences within phenotypically homogeneous populations become more sophisticated. Also, the fate of memory lymphocytes that are restimulated regularly by pathogens is of particular relevance to human immunology. Such questions have proven extremely challenging, given the difficulty in following populations of memory cells across time and through multiple challenges. At a population level, studies have suggested that the memory compartment can increase in size substantially. Tellingly, many human tissues have substantial populations of memory lymphocytes (5). However, the fate of memory cells that are restimulated multiple times is not clear, either at the population or single cell level. Since chronic antigenic stimulation can negatively impact on lymphocyte functions (13), it will be valuable to determine whether memory cells induced by infection or vaccination, which are repeatedly restimulated, retain the ability to quickly and efficiently deal with infections, and if not how we can overcome this.

Another question highly relevant to the field of immunological memory is whether innate immune cells also acquire traits of memory cells after challenge (14). This is established for NK cells that can elicit antigen-specific memory functions in response to viral infection. It is less clear whether other innate lymphoid cell lineages will show similar traits. It is feasible that, like NK cells, many innate cells can be imprinted non-specifically by epigenetic mechanisms to elicit distinct responses upon recall. Whether this constitutes immunological memory will then need to be defined by the field.

The translational challenge that lies ahead is an ability to design new vaccines that can control infections and cancers, within highly diverse human populations. Though we have learnt much about how immune memory populations are formed and can function to protect against disease, the application of this knowledge to many pathogens remains elusive. The recent advances in the field of immunological memory provide plenty of scope for many of these questions to be answered in the near future.

## Author Contributions

SM conceived and wrote the article.

## Conflict of Interest Statement

The author declares that the research was conducted in the absence of any commercial or financial relationships that could be construed as a potential conflict of interest.
